# AI-augmented pathology: the experience of transfer learning and intra-domain data diversity in breast cancer metastasis detection

**DOI:** 10.3389/fonc.2025.1598289

**Published:** 2025-06-11

**Authors:** Manuel Cossio, Nina Wiedemann, Esther Sanfeliu Torres, Esther Barnadas Sole, Laura Igual

**Affiliations:** ^1^ Department of Mathematics and Computer Science, Universitat de Barcelona, Barcelona, Spain; ^2^ Institute of Cartography and Geoinformation, ETH, Zürich, Zürich, Switzerland; ^3^ Biomedical Diagnostic Center, Clinic Hospital, Barcelona, Spain

**Keywords:** sentinel lymph node, breast cancer, metastasis detection, digital pathology, transfer learning

## Abstract

**Background:**

Metastatic detection in sentinel lymph nodes remains a crucial prognostic factor in breast cancer management, with accurate and timely diagnosis directly impacting treatment decisions. While traditional histopathological assessment relies on microscopic examination of stained tissues, the digitization of slides as whole-slide images (WSI) has enabled the development of computer-aided diagnostic systems. These automated approaches offer potential improvements in detection consistency and efficiency compared to conventional methods.

**Results:**

This study leverages transfer learning on hematoxylin and eosin (HE) WSIs to achieve computationally efficient metastasis detection without compromising accuracy. We propose an approach for generating segmentation masks by transferring spatial annotations from immunohistochemistry (IHC) WSIs to corresponding H&E slides. Using these masks, four distinct datasets were constructed to fine-tune a pretrained ResNet50 model across eight different configurations, incorporating varied dataset combinations and data augmentation techniques. To enhance interpretability, we developed a visualization tool that employs color-coded probability maps to highlight tumor regions alongside their prediction confidence. Our experiments demonstrated that integrating an external dataset (Camelyon16) during training significantly improved model performance, surpassing the benefits of data augmentation alone. The optimal model, trained on both external and local data, achieved an accuracy and F1-score of 0.98, outperforming existing state-of-the-art methods.

**Conclusions:**

This study demonstrates that transfer learning architectures, when enhanced with multi-source data integration and interpretability frameworks, can significantly improve metastatic detection in whole slide imaging. Our methodology achieves diagnostic performance comparable to gold-standard techniques while dramatically accelerating analytical workflows. The synergistic combination of external dataset incorporation and probabilistic visualization outputs provides a clinically actionable solution that maintains both computational efficiency and pathological interpretability.

## 1 Background

Breast cancer is the most commonly diagnosed cancer among women worldwide, posing a significant global health burden (1). The prognosis of breast cancer patients is closely tied to the stage of disease progression, with survival rates declining sharply upon the development of distant metastases. As a result, early detection is critical to improving therapeutic outcomes and patient survival (2, 3).

**Figure 1:**
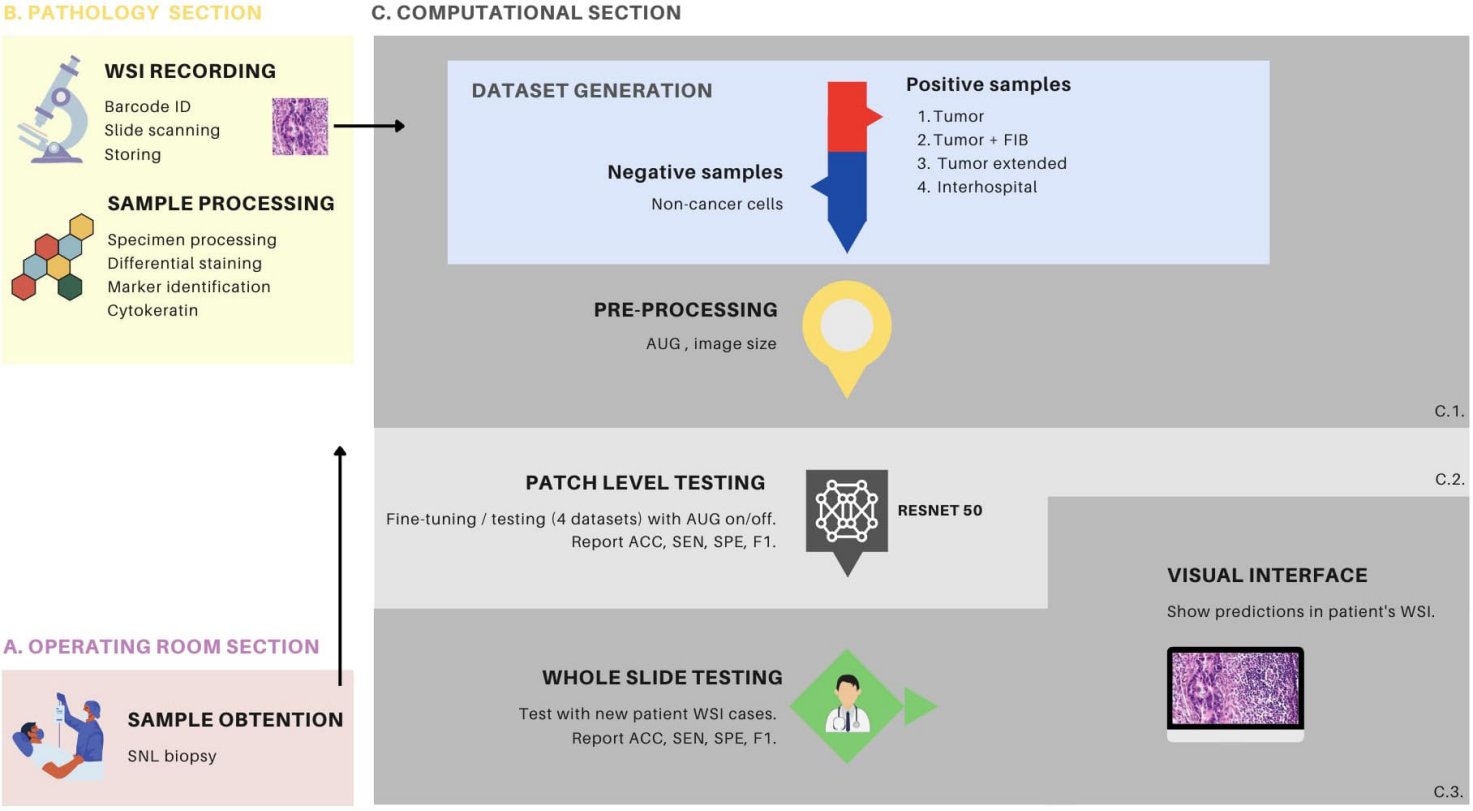
Overview of the metastasis detection process with transfer learning. **(A)** demonstrates the sampling process conducted in the operating room, wherein the surgeon performs a procedure to locate and extract the sentinel lymph node (SLN) for subsequent pathology processing. **(B)** depicts the biological sample processing and image recording stages, which comprises two primary components. The first component involves slide preparation, hematoxylin and eosin (HE) staining, and immunohistochemistry (IHC) with cytokeratin staining. The second component involves slide identification via a barcode, image scanning as a whole slide image (WSI) file, and storage within the hospital server. **(C)** the computational section, comprises three distinct components. C1 pertains to the procedures associated with data generation and data processing, C2 is dedicated to the fine-tuning of the ResNet50 model, and the patch-level testing thereof, while C3 is focused on whole-slide testing. C1, in particular, encompasses the generation of 4 distinct patch datasets and encompasses the data preprocessing stage, which includes the creation of patches and various data augmentation techniques. C2 is characterized by the detailed procedures for fine-tuning the ResNet50 model, employing various configurations, including the different dataset types and augmentation settings. In this phase, patch-level testing is performed, and metrics such as accuracy (ACC), sensitivity (SEN), specificity (SPE), and F1-score (F1) are reported. C3 provides an elaborate account of the patch-level testing, using patient WSI of HE stained tissue sections. This phase also involves the reporting of metrics such as ACC, SEN, SPE and F1. Furthermore, it elucidates the implementation of a visual interface designed for medical professionals to facilitate the interpretation of model predictions. This interface supports the analysis and assessment of the model’s performance, thereby enhancing its practical utility in clinical settings. FIB, tumor associated fibrosis; AUG, augmentations.

The evaluation of metastatic status in breast cancer patients involves both preoperative and postoperative assessments. Postoperative analyses provide detailed information about tumor characteristics, including lymphatic and vascular invasion, the extent of necrosis, and the degree of epithelial hyperplasia (4). While these parameters are valuable indicators of metastatic potential, they are only available after surgical intervention. Preoperative diagnostic tools, such as imaging modalities, offer preliminary insights into tumor behavior. However, definitive assessment of lymph node involvement—a key determinant of metastatic spread—relies on the analysis of the sentinel lymph node (SLN) obtained during surgery (5, 6) (**Figure 1A**). SLN biopsy is a minimally invasive technique that enables early detection of tumor cells disseminated from the primary site, facilitating precise therapeutic planning and reducing the need for more extensive surgical procedures (4, 5).

**Figure 2:**
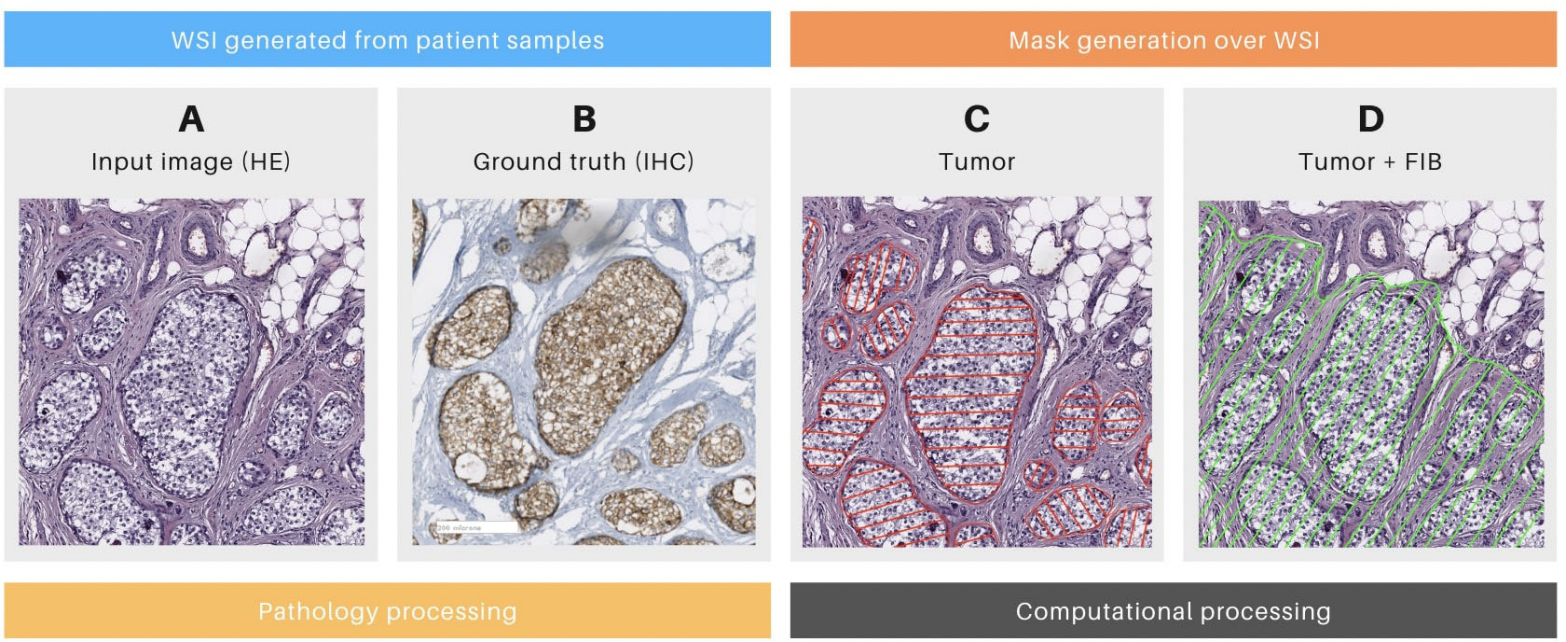
Details of the mask generation process. **(A)** Patch of a whole slide image (WSI) stained with hematoxylin and eosin (HE) with tumoral cells. **(B)** The consecutive slice of the same surgical piece, stained with Cytokeratin immunohistochemistry (IHC, brown deposits indicating tumoral cells). **(C)** Mask generated only over tumoral cells. **(D)** Mask generated over tumoral cells and tumor-associated fibrosis (FIB). **(A, B)** were generated during the pathology section. **(C, D)** Were generated during the computational section.

Following SLN biopsy, the tissue is processed and evaluated by pathologists through microscopic examination. Tissue structures are differentiated using staining techniques, such as hematoxylin and eosin (HE) for general morphology or immunohistochemistry (IHC) for specific biomarkers (7) (**Figures 2A, B**, respectively). IHC is particularly valuable for identifying metastatic cells within normal parenchyma, especially in cases where morphological differentiation between normal and abnormal tissue is challenging using HE staining alone (8, 9) (**Figure 1B**). The IHC process involves the use of primary antibodies that bind to specific antigens in the tissue, followed by enzyme-conjugated secondary antibodies that produce a visible signal, typically a brown precipitate, at the target site (10).

Advances in medical technology have enabled the digitization of histopathological slides, resulting in whole-slide images (WSIs). WSIs capture multiple magnifications of the same tissue region within a single file, allowing pathologists to navigate seamlessly from a macroscopic overview to high-resolution details. This digital transformation has facilitated the application of computer vision algorithms, particularly convolutional neural networks (CNNs), to automate the detection of tumor regions in biopsy specimens. Researchers have developed CNN-based models capable of identifying various cancer types, including breast cancer, within WSIs (11–17). These models are trained on annotated datasets containing both tumor and healthy tissue samples, enabling them to learn discriminative patterns for accurate classification (18). To manage the computational demands of processing large WSIs, images are often divided into smaller patches at the highest resolution. The CNN generates patch-wise predictions, which are subsequently aggregated to produce comprehensive diagnostic visualizations (19) (**Figure 1C**).

Despite its potential, computational pathology faces two key challenges. First, the limited availability of patient data often requires multi-institutional collaborations, delaying research progress (20). Second, the computational resources needed for training models are immense, with studies requiring terabytes of storage and hundreds of thousands of GPU hours (21). These demands strain infrastructure and contribute to environmental concerns, as medical imaging accounts for approximately 1% of global greenhouse gas emissions (22).

This study proposes a transfer learning approach to address critical challenges in computational pathology for metastatic tumor detection. Building on the established success of deep learning in medical imaging, we investigate whether pretrained models can be effectively adapted to overcome data scarcity and computational constraints in clinical settings. Our work is guided by three principal hypotheses: First, that transfer learning with strategic fine-tuning can extract diagnostically relevant patterns from limited histopathology datasets while maintaining clinical-grade accuracy. Second, combining institutional data with curated public repositories will yield more robust feature representations than either source alone. Third, integrating intuitive visual interpretability tools can render model decisions transparent to pathologists without requiring specialized technical expertise.

The experimental framework employs a ResNet50 architecture initialized with ImageNet weights, subsequently fine-tuned on annotated whole-slide images of sentinel lymph node biopsies from Hospital Clinic Barcelona^1^. Through carefully designed comparisons across eight experimental conditions, we systematically evaluate the impact of key factors including: (i) data composition strategies balancing local and public datasets, (ii) augmentation techniques for improved generalization, and (iii) evaluation protocols that assess both computational and clinical viability.

A novel aspect of this research is its dual-focus evaluation methodology, which combines traditional performance metrics with clinician-centric visualization tools. This approach seeks to establish whether computational predictions can simultaneously satisfy quantitative benchmarks and operational requirements for pathological diagnosis. The study design intentionally avoids presupposing optimal configurations, instead examining how varying degrees of data diversity and explainability influence model behavior in diagnostically relevant scenarios.

## 2 Related work

The detection of metastasis from primary tumors is a critical step in tumor staging, with SLN biopsies serving as the gold standard for assessing lymph node involvement (5, 6). Historically, pathologists relied on optical microscopes to examine processed and stained tissue samples (23). However, the introduction of digital slide scanners has transformed this process, enabling the creation and storage of WSIs that can be analyzed computationally (24).

A central challenge in automated WSI analysis is the accurate classification of tumor regions. This requires the generation of masks—annotations that distinguish cancerous cells from healthy tissue. Masks can be binary, indicating the presence or absence of cancer, or multi-label, providing additional information such as malignancy grades or the presence of immune cells within the tumor microenvironment (25, 26). These masks are typically created by pathologists or derived from ICH techniques. In the latter approach, IHC is used to identify cancer regions in a tissue slice adjacent to the HE-stained slide. The spatial information from the IHC slice is then transferred to the HE slide to generate the corresponding mask (26, 27).

In machine learning-based analysis of WSIs, the images are typically divided into smaller patches, where patch size serves as a critical parameter affecting both computational efficiency and the model’s capacity to capture relevant contextual information (19, 28, 29). To mitigate challenges associated with limited dataset size and variability, data augmentation techniques—including rotation, contrast adjustment, noise injection, and grayscale conversion—are widely adopted (15, 16, 30, 31). While such augmentations can enhance model robustness and generalization, excessive application may introduce artificial patterns or distort underlying data distributions (32). Notably, some studies mention some benefits of augmentation without providing full comparative analyses against non-augmented baselines, raising questions about their necessity in certain contexts (33).

Two primary methodologies are commonly employed for training models in WSI patch classification. The first is transfer learning, wherein a model pretrained on a large-scale dataset (e.g., ImageNet) is fine-tuned for the target histopathological task. A key variant of this approach is end-to-end learning, which unifies feature extraction and classification within a single framework, enabling direct learning from image patches. This method is particularly beneficial when working with limited annotated data, as it leverages pre-existing feature representations. Widely used architectures for transfer learning include ResNet, AlexNet, DenseNet, EfficientNet, Inception V3, and VGG (34–37). For example, ResNet-50 has demonstrated strong performance in breast cancer histopathology detection (38). However, a notable limitation of these deep learning models is their inherent “black-box” nature, often lacking interpretability in decision-making processes.

The second methodology separates feature extraction from classification. Handcrafted feature extraction involves using domain specific knowledge and algorithmic methods to identify and quantify relevant features within an image. These features are then fed into a separate classifier, such as a Support Vector Machine (SVM) or Random Forest (34, 39, 40). Examples of handcrafted features include those derived from texture analysis (e.g., Local Binary Patterns, Gabor filters), edge detection (e.g., Canny edge detector), or feature descriptors (e.g., SIFT, HOG) (41–43). While handcrafted features are interpretable, they require extensive domain expertise and may not capture high-level abstractions as effectively as deep learning models.

The performance of deep learning models in histopathology is typically evaluated using a combination of quantitative metrics and qualitative visualizations. Standard quantitative measures include accuracy, sensitivity, specificity, precision, recall, F1-score, and the Area Under the Receiver Operating Characteristic Curve (AUC-ROC) (19, 28, 40). On the qualitative side, visual assessments often involve the use of heatmaps, which highlight regions of interest within WSIs. These heatmaps can be displayed independently or overlaid on the original tissue images, occasionally requiring grayscale conversion to improve visual clarity (28, 44). Such visualizations play a critical role in aiding medical professionals by enhancing the interpretability of model predictions and by facilitating the identification of cancerous regions, which in turn can support the development of new training datasets (28).

Among the various visualization techniques, Class Activation Map (CAM)-based methods are widely used for explaining CNN outputs. These techniques work by projecting back the weights from the final convolutional layers to generate saliency maps that highlight spatial regions most influential to a model’s prediction (45). While effective for small-scale images, applying CAM-based methods to WSIs—which can exceed 10 billion pixels at high resolution—poses significant challenges. First, generating maps for each of the thousands of patches in a WSI can be computationally expensive. Second, the resulting pixel-level heatmaps across such a massive canvas can become visually overwhelming, introducing excessive detail that obscures broader diagnostic patterns. Third, in contrast to natural images where objects of interest are often visually prominent, regions of interest in pathology images tend to be subtle and lack strong saliency or clear boundaries. This makes it difficult for CAM-based methods to consistently localize relevant features, often resulting in under- or over-activation of regions and reducing the interpretability of the visualization at the clinical scale (45–47).

In summary, despite notable advancements in automated WSIs analysis, key challenges persist in addressing data variability, optimizing data augmentation, and improving model interpretability. Our work advances current methodologies by (1) incorporating external datasets to better capture domain variability, (2) systematically evaluating the influence of augmentations on model performance, and (3) developing visualization tools to increase the transparency of deep learning models in metastasis detection.

## 3 Data and methods

### 3.1 Data

In this study, WSIs were obtained from the Pathological Anatomy Service of the Biomedical Diagnostic Center at the Hospital Clinic Barcelona (BDC-HCB), Barcelona. The images were digitized using the Roche Diagnostics Ventana DP200 slide scanner at a final magnification of 40X. To ensure data privacy, the images were stored on a secure virtual disk with encryption and password-protected access. The Ethics Committee of HCB authorized the use of the images for this study.^2^ BDC-HCB provided two distinct batches of images. The first batch comprised 10 hematoxylin and eosin (HE) images and 5 immunohistochemical (IHC) images, which were utilized for model fine-tuning and patch-based testing. These images had an average resolution of 0.5 µm/px and an average size of 75,000 px × 50,000 px. Among the HE images, five were confirmed as positive for metastasis in the sentinel lymph node (SLN) (**Table 1**, columns HE’+’), as validated by IHC using the mouse monoclonal antibody Cytokeratin 19 (A53-B/A2.26) against simple and complex epithelia, developed by Cell Marque Tissue Diagnostics (**Table 1**, columns IHC-GT).

**Table 1:** Outline of the patch dataset generation procedure concerning image acquisition origins.

WSI						Patches Production				Patch Datasets	
HE (+)		HE (-)		IHC-GT		Creation		Label			
Origin	WSI	Origin	WSI	Origin	WSI	MC	Size	(+)	(-)	ID	PN
1. HCB	n = 5	HCB	n = 5	HCB	n = 5	YES	90x90	TC >20%	TC = 0	Tumor	23,900
2. HCB	n = 5	HCB	n = 5	HCB	n = 5	YES	90x90	TC >20%	TC = 0	Tumor + FIB	23,900
3. HCB	n = 5	HCB	n = 5	HCB	n = 5	YES	90x90	TC >20%	TC = 0	Tumor Extended	227,923
4. HCB	n = 5	HCB	n = 5	HCB	n = 5	YES	90x90	TC >20%	TC = 0	Interhospital	104,107
5. CAM16	n = 5	0	0	0	0	NO	90x90	TC >20%	TC = 0		

The presented table provides a comprehensive enumeration of whole slide image (WSIs) processing steps for the creation of specific patch datasets. Each row in the table corresponds to a distinct patch dataset and outlines various essential parameters. The “WSI” columns denote the source of the WSI to create patches, distinguishing between Hospital Clinic Barcelona (HCB) and Camelyon 16 (CAM). Within the table, “HE +” signifies the count of positive hematoxylin and eosin (HE) WSI, “HE -” represents the count of negative HE WSI, and “IHC-GT” indicates the number of immunohistochemistry WSI featuring brown deposits used as ground truth (GT) references. Utilizing the information from IHC-GT, masks for HE + patches are constructed, denoted in the mask creation “MC” column with a value of YES. CAM WSIs come pre-equipped with accompanying masks that have been published alongside the images, therefore the “MC” column is designated as NO. The “Size” column provides the dimensions of the resultant patches, while the “Label” column indicates the tumor content (TC) necessary for classifying a patch as positive or negative. The “ID” column specifies the corresponding patch dataset identification, and the “PN” column enumerates patch number. Notably, the ‘Interhospital’ patch dataset, as delineated in rows 4 and 5, amalgamates data from both HCB and CAM sources, consolidating patches into a unified dataset. FIB, tumor associated fibrosis.

Consequently, each positive case included two corresponding images: one in bright field HE and another in bright field IHC. A key diagnostic feature of cancer cells in IHC images is the presence of brown deposits. The remaining five HE images, which were negative for SLN metastasis, were provided exclusively in bright field HE format (**Table 1**, columns HE’-’). The second batch, consisting of 5 positive HE images and 5 corresponding IHC images, was reserved for whole slide testing.

Following the pathology section, the computational section was initiated, beginning with the annotation process. Tumor masks were generated by manually delineating tumor regions in HE images from positive cases (**Figures 2C, D**; **Table 1**, column MC). Tumor areas were identified based on the presence of the marker in the corresponding IHC WSIs, as confirmed by pathologists (**Figure 2B**). The delineation of these masks was performed using the *labelme* ^3^ software, applied to each previously identified tumor region. Each mask assigned a value of 1 to pixels corresponding to tumor cells and 0 to non-tumor cells. The alignment of the masks was rigorously validated through a three-stage visual inspection process: first by the pathologist, then by the imaging technician, and finally by the computational engineer.

Patches measuring 90 × 90 pixels were extracted from the WSIs, each containing regions labeled as positive (tumor), negative (non-tumor), or a mix of both. A patch was classified as positive if more than 20% of its pixels were tumor-associated (**Table 1**, column Label ‘+’). Critically, no upper threshold was imposed—patches with varying tumor burdens (e.g., 30%, 50%, 80%, etc.) were randomly included in the positive class to improve model generalizability and reduce the risk of misclassifying partially tumorous patches. Conversely, patches with no tumor pixels were labeled as negative (**Table 1**, column Label ‘-’). To address class imbalance, the more abundant negative class was subsampled to match the number of positive patches. Finally, a subset of patches was manually reviewed to verify label accuracy.

**Figure 3:**
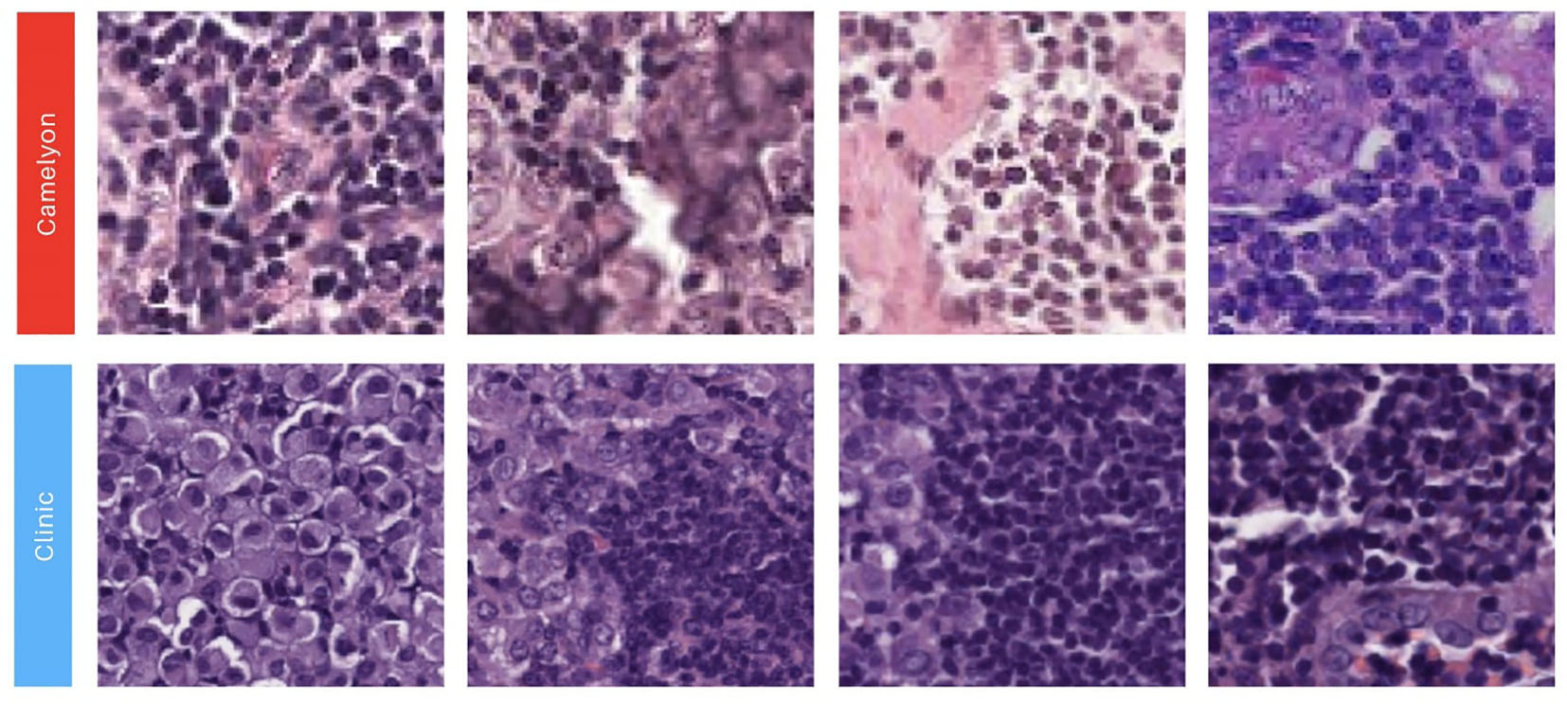
Illustration showcasing patches extracted from our two image sources. The first row showcases 90x90 image patches with a positive label obtained from the Camelyon 16 dataset (3, 48). The second row presents 90x90 positive image patches sourced from the Biomedical Diagnostic Center of the Hospital Clinic (BDC-HC) in Barcelona.

Four patch datasets were constructed using the first batch of WSIs, which included 10 HE images (5 positive and 5 negative cases), for model training (**Table 1**, columns Patch datasets). The first dataset, labeled ‘Tumor’, exclusively contained patches with tumor cells, as defined by the masks of positive patches (**Table 1**, first row). The second dataset, ‘Tumor + FIB’, incorporated tumor-related fibrosis alongside tumor cells within the masks of its positive patches (**Table 1**, second row). The third dataset, ‘Tumor extended’, was created by expanding the ‘Tumor’ dataset with additional patches (**Table 1**, third row). The fourth dataset, ‘Interhospital’, comprised a 50%/50% mixture of patches from the ‘Tumor’ dataset and samples from the Camelyon 16 dataset of equivalent size (**Table 1**, fourth row). The Camelyon 16 dataset was developed by retrospectively sampling sentinel lymph nodes (SLNs) from 399 patients who underwent breast cancer surgery at two Dutch hospitals: Radboud University Medical Center (RUMC) and University Medical Center Utrecht (UMCU). The slides were digitized using a Pannoramic 250 Flash II scanner (3DHISTECH) with a 20x objective lens. Metastases on the slides were annotated under the supervision of expert pathologists. While obvious metastases were annotated without IHC staining, IHC (anti-Cytokeratin [CAM 5.2], BD Biosciences) was employed for ambiguous cases (3, 48). No image normalization techniques—such as histogram equalization or style transfer—were applied to the Camelyon16 dataset, as its inherent variability was intentionally preserved to assess its impact on model performance. As shown in **Figure 3**, noticeable differences in contrast and saturation are evident across samples from different sources.

### 3.2 Data augmentation and preprocessing

The image preprocessing pipeline incorporated multiple data augmentation techniques to enhance model robustness. First, color space transformations were applied by converting images to grayscale followed by transformation from RGB to HSV color space, with saturation values randomly sampled from a uniform distribution between 1 and 180. Noise augmentation included two components: Gaussian noise with mean values randomly selected from 0 to 10, and salt-and-pepper noise applied with probabilities ranging uniformly from 0 to 1. Additional image manipulations consisted of RGB channel separation and contrast adjustment through random scaling factors *α* uniformly distributed between 0 and 10, combined with brightness modification using random offsets *β* sampled from 0 to 10. Finally, all images underwent normalization to the (0,1) range, with subsequent calculation of per-channel mean and standard deviation values across the entire dataset.

### 3.3 Deep neural network model

We employed a pre-trained ResNet-50 architecture, implementing a transfer learning strategy that has demonstrated effectiveness in both general image classification tasks (49) and specialized medical imaging applications including breast cancer pathology detection (39, 50). The ResNet-50 model consists of five hierarchical stages, each containing one convolution block and one identity block, with every block comprising three convolutional layers. This architecture, containing approximately 23 million trainable parameters (51), has proven particularly successful in cancer pathology detection tasks as evidenced by previous studies (52, 53).

The model initialization utilized weights pre-trained on the ImageNet dataset (1000 classes), followed by task-specific fine-tuning for our histopathology application. All training experiments were conducted in a Google Colaboratory environment^4^ with NVIDIA Tesla K80 GPU acceleration. During fine-tuning, we adopted a strategic approach where the first three convolutional layers remained frozen to preserve their pre-trained weights, while all subsequent layers including the classification head were made trainable. This design allowed the model to maintain robust low-level feature extractors while adapting higher-level features to our specific histopathology domain.

The training protocol employed cross-entropy loss function with a batch size of 128 across 18 epochs. We implemented weight decay (L2 regularization) with a coefficient of 1 × 10^−4^ and used an adaptive learning rate schedule with an initial maximum value of 2 × 10^−2^ that followed a cosine annealing pattern. This combination of hyperparameters was carefully selected to balance efficient convergence with effective regularization, enabling the model to adapt its higher-level representations while maintaining the fundamental visual feature extractors learned from the large-scale ImageNet dataset.

### 3.4 Experimental setup

#### 3.4.1 Patch-based testing

For patch-based testing, eight fine-tuning experiments were conducted, each designed to address specific training conditions as described in **Table 2**. These experiments involved four distinct patch datasets, with two experiments performed per dataset: one using original, unaltered patches and the other utilizing augmented patches (see **Table 2**). The data were split into training and testing sets using a 70/30 ratio. After training and testing, the model weights were saved and exported, resulting in a total of eight preserved models. Additionally, validation measures were computed, as outlined in section 3.4.3.

**Table 2:** Comprehensive summary of the experiments conducted to fine-tune and evaluate a ResNet50 model for sentinel lymph node biopsy classification.

Fine-tuning			Patch-based Testing	Whole Slide Testing	
Experiment	Dataset	Augmentation	Metrics across models (T4)	Metrics across models (T5)	Metrics across WSI (T6) 1
1	Tumor	0	ACC, SEN, SPE, F1 (Mean)	ACC, SEN, SPE, F1 (Mean ± SD)	ACC, SEN, SPE, F1 (Mean ± SD)
2	Tumor	1	ACC, SEN, SPE, F1 (Mean)	ACC, SEN, SPE, F1 (Mean ± SD)	ACC, SEN, SPE, F1 (Mean ± SD)
3	Tumor + FIB	0	ACC, SEN, SPE, F1 (Mean)	ACC, SEN, SPE, F1 (Mean ± SD)	ACC, SEN, SPE, F1 (Mean ± SD)
4	Tumor + FIB	1	ACC, SEN, SPE, F1 (Mean)	ACC, SEN, SPE, F1 (Mean ± SD)	ACC, SEN, SPE, F1 (Mean ± SD)
5	Tumor Extended	0	ACC, SEN, SPE, F1 (Mean)	ACC, SEN, SPE, F1 (Mean ± SD)	ACC, SEN, SPE, F1 (Mean ± SD)
6	Tumor Extended	1	ACC, SEN, SPE, F1 (Mean)	ACC, SEN, SPE, F1 (Mean ± SD)	ACC, SEN, SPE, F1 (Mean ± SD)
7	Interhospital	0	ACC, SEN, SPE, F1 (Mean)	ACC, SEN, SPE, F1 (Mean ± SD)	ACC, SEN, SPE, F1 (Mean ± SD)
8	Interhospital	1	ACC, SEN, SPE, F1 (Mean)	ACC, SEN, SPE, F1 (Mean ± SD)	ACC, SEN, SPE, F1 (Mean ± SD)

The table presents a two-pronged analytical approach for assessing the performance of eight ResNet50 models, initially fully trained on the ImageNet dataset, in classifying sentinel lymph node (SLN) status. The models underwent a series of fine-tuning experiments (column 1) using different datasets (column 2), with the 10 application of data augmentation assessed in a binary fashion (column 3). Performance metrics, Accuracy (ACC), Sensitivity (SEN), Specificity (SPE), and weighted F1 score, were evaluated under two testing conditions. The ’Patch-based Testing’ (column 4) determined the efficacy of models using patches from the same whole slide images (WSIs) used for fine-tuning, while the ’Whole Slide Testing’ (columns 5 and 6) involved a comprehensive assessment with unseen WSI, first by collating the aforementioned metrics across identical models (column 5) for each WSI (16, 18, 23, 25, and 27) and computing the mean and standard deviation (SD), then by aggregating these metrics for each WSI across all models (column 6), also calculating mean and SD for a thorough inter-model and intra-WSI comparison. This dual analysis elucidates both the collective and individual model performances relative to each WSI, offering a granular view of model robustness and diagnostic precision. T4, T5 and T6 refer to **Tables 4**, **5** and **6** containing reported results.

#### 3.4.2 Whole slide testing

To assess the robustness of the models generated in each experiment, we performed whole slide testing. This involved making predictions on WSIs that were not used for generating training patches. Specifically, the models were tested on a set of 5 WSIs from new cancer patients, each containing varying percentages of cancer pixels (see **Table 3**, column 1). Notably, these WSIs were entirely unseen by any of the models during training. To summarize the results, we generated tables reporting the mean and standard deviation (SD) of model performance. These tables were organized either by experiment or by patient WSI (refer to **Table 2**, columns 5 and 6). When results were grouped by model, the mean and SD were calculated across all five patients. Conversely, when grouped by patient, the results were averaged across all four trained models. This final analysis enabled us to identify the patients for whom the models encountered the most significant performance challenges.

**Table 3:** Details of pixel content in whole slide testing samples.

Case	HE	Case	IHC	Tissue (%)	Tumor (%)
16	1	17	1	99.97	0.03
18	1	19	1	99.65	0.35
23	1	24	1	70.59	29.41
25	1	26	1	81.36	18.64
27	1	28	1	94.89	5.11

Percentage of tissue and tumor pixels per whole slide image (WSI) are detailed in the last columns.

#### 3.4.3 Validation measures

**Figure 4:**
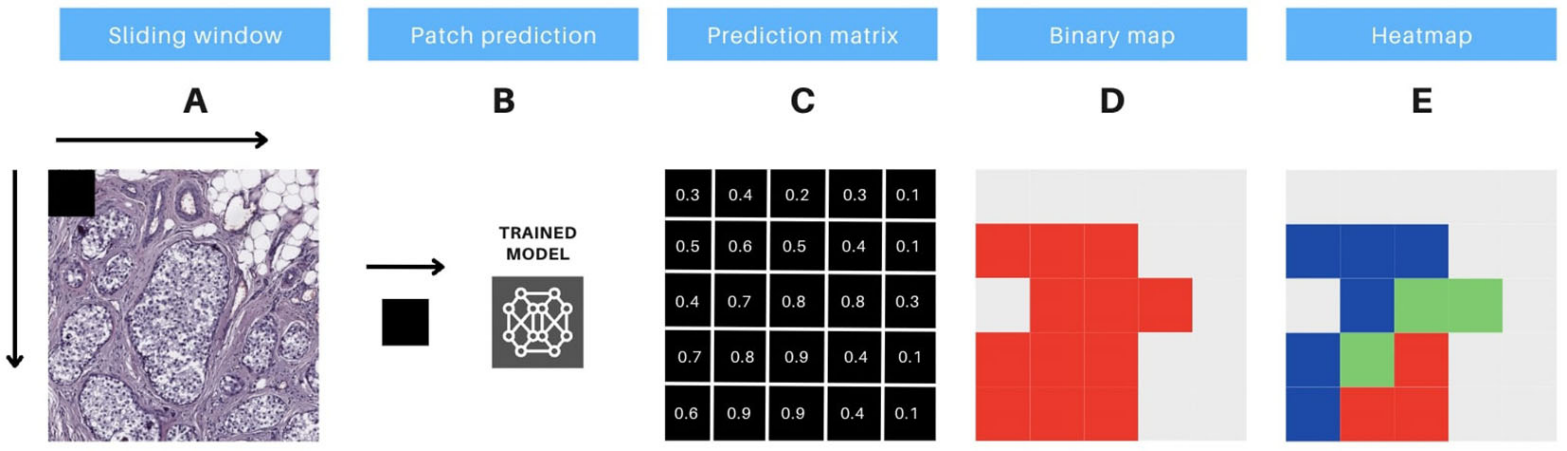
Protocol for prediction interpretation and visual aid. The methodology includes 5 steps, as follows: **(A)** the sliding window technique is applied to extract consecutive non-overlapping patches of 90x90 pixels from a whole slide image (WSI). **(B)** these image patches are then input to a pre-trained model, which generates predictions for each patch. **(C)** the output of the model generates a prediction matrix, where each prediction is assigned to the corresponding location of the patch. **(D)** a binary color map is utilized to visualize the predicted values above the threshold of 0.5, with red indicating the patches that meet this criterion. **(E)** a heatmap is constructed, utilizing red, green, and blue colors, to display the predicted probabilities for each patch. The color codes represent high (above 0.9), medium (0.7 to 0.9), and low probability (0.5 to 0.7) predictions, respectively.

To thoroughly evaluate the performance of the trained models in each experiment, we first generated confusion matrices for the patch-based testing stage. These matrices were created using Fastai’s built-in functionality, with text-format labels assigned to each patch. For the whole slide testing stage, we implemented a custom sliding window method (illustrated in **Figure 4**). This method utilized entire WSIs as the source of patches, with a binary mask serving as the ground truth reference. The output of this process was a probability matrix, where the prediction for each patch was spatially linked to its neighboring patches (see **Figure 4C**).

To classify patches as positive or negative, a threshold of 0.5 was applied. Patches with probabilities above this threshold were classified as positive, while those below were classified as negative. By comparing the patches with their corresponding labels in the binary mask, we identified true positives (TP) and true negatives (TN) where the labels matched. Patches with mismatched labels were classified as false positives (FP) or false negatives (FN).

We then calculated several validation metrics to assess model performance. These included accuracy (ACC) for analytical validation, sensitivity (SEN), specificity (SPE), and weighted F1-score (F1) for clinical validation, in accordance with the International Medical Device Regulator Forum’s (IMDRF) guidelines for the clinical evaluation of Software as a Medical Device (SaMD)^5^. Additionally, AUC-ROC curves were constructed to further evaluate model performance.

Accuracy was calculated as the ratio of correctly classified instances (TP + TN) to the total number of instances (TP + TN + FP + FN). Sensitivity was determined as the ratio of TP to the sum of TP and FN. Specificity was computed as the ratio of TN to the sum of TN and FP. The F1 score, which combines precision (TP/(TP + FP)) and recall (sensitivity), was derived as the harmonic mean of these two metrics, providing a balanced measure of model performance.


$$\text{Accuracy}=\frac{TP+TN}{TP+TN+FP+FN}$$

$$\text{Sensitivity}\;\left(\text{Recall}\right)=\frac{TP}{TP+FN}$$

$$\text{Specificity}=\frac{TN}{TN+FP}$$

$$\text{Precision}=\frac{TP}{TP+FP}$$

$$\text{F}1\;\mathrm{score}=2\times \frac{\text{Precision}\times \text{Recall}}{\text{Precision}+\text{Recall}}=\frac{2TP}{2TP+FP+FN}$$

#### 3.4.4 Visual aid for classification interpretation

To enhance prediction interpretability and foster collaboration between physicians and data scientists, we developed a visual aid system based on tumor classification prediction matrices, featuring two visual tools. This system directly visualizes model predictions on image patches, avoiding excessive detail when composing the complete WSI, and bypasses further computations on model weights.

The first tool is a high-contrast monochromatic map that highlights all regions of the image with a tumor prediction probability above 0.5 (as illustrated in **Figure 4D**). The second tool is a heatmap that uses three distinct colors to differentiate patches based on their prediction probabilities (as shown in **Figure 4E**). The color-coding for the heatmap is defined as follows: red for high-probability predictions (above 0.9), green for medium-probability predictions (between 0.7 and 0.9), and blue for low-probability predictions (between 0.5 and 0.7). These visual tools are designed to enhance the clarity of the model’s predictions and support effective communication between healthcare professionals and data scientists.

## 4 Results

In this section, we present the results of our experiments, as summarized in **Table 2**. Initially, we computed histograms for the 4 patch datasets generated. **Supplementary Figure 1** illustrates that the histograms for positive samples in all 4 patch datasets exhibited a similar distribution to those of the negative samples, confirming an expected increase in the relative frequency of pixels with value 255 due to white patches in negative samples.

In the subsequent fine-tuning and patch-based testing phase, both processes utilized patches derived from the same WSIs. As detailed in **Table 4**, optimal performance across all models was consistently achieved when fine-tuning was performed without the application of data augmentations. Specifically, the ‘Interhospital’ model (Experiment 7) demonstrated the highest accuracy (0.881), while the ‘Tumor + FIB’ model (Experiment 3) exhibited the best sensitivity (0.756) and weighted F1-score. Notably, the ‘Tumor’ model (Experiment 1) achieved the highest specificity (0.987). Regarding computational efficiency, model training times, conducted using NVIDIA Tesla K80 hardware for a fixed 18 epochs per dataset, varied considerably, and this variation appears correlated with dataset size. The ‘Tumor’ and ‘Tumor + FIB’ datasets, comprising 23,900 samples each, showed the most rapid training, both completing in approximately 5.87 minutes. In contrast, the ‘Interhospital’ dataset, with 104,107 samples, required a significantly longer duration of 25.55 minutes, and the ‘Tumor extended’ dataset, the largest with 227,923 samples, presented the longest training time at 55.93 minutes.

**Table 4:** Mean of patch-based testing metrics for each model with and without augmentations.

Model	AUG	Testing			
		ACC	SEN	SPE	F1
Tumor	0 1	**0.673** 0.671	0.232 0.232	**0.987** 0.983	**0.856** 0.843
Tumor + FIB	0 1	**0.878** 0.729	**0.756** 0.425	**0.970** 0.956	**0.913** 0.865
Tumor extended	0 1	**0.477** 0.467	0.001 0.001	**0.973** 0.968	**0.723** 0.695
Interhospital	0 1	**0.881** 0.787	0.000 0.000	**0.948** 0.895	**0.890** 0.810

FIB, tumor related fibrosis; AUG, augmentations; ACC, accuracy; SEN, sensitivity; SPE, specificity; F1, weighted F1-score. Bold values indicate the highest value of the 2 metrics per model (with and without AUG).

Following the fine-tuning phase, our research extended to the whole slide testing phase (**Table 2**, Whole Slide Testing). The metrics obtained during this phase differed significantly from the preliminary patch-based testing metrics (**Table 4**). To capture these differences, two tables were generated. **Table 5** outlined the results for each fine-tuned model with and without augmentations, while **Table 6** detailed the results for each image (individual patient) across all models, both with and without augmentations. These analyses aimed to identify robust models across all images and assess the difficulty levels in detecting metastasis for each image using all models.

**Table 5:** Mean and standard deviation of whole slide testing metrics for each model with and without augmentations.

Model	AUG	ACC		SEN		SPE		F1	
		Mean	SD	Mean	SD	Mean	SD	Mean	SD
Tumor	0 1	0.978 0.978	0.019 0.015	0.001 **0.106**	0.002 0.055	**0.995** 0.994	0.008 0.008	0.971 **0.975**	0.029 0.023
Tumor + FIB	0 1	**0.979** 0.978	0.020 0.014	0.001 **0.241**	0.002 0.132	0.991 **0.992**	0.004 0.002	0.972 **0.977**	0.029 0.021
Tumor extended	0 1	0.977 **0.981**	0.020 0.019	**0.005** 0.000	0.009 0.000	0.995 **0.998**	0.010 0.002	0.971 **0.973**	0.028 0.029
Interhospital	0 1	0.958 **0.982**	0.050 0.015	0.219 **0.256**	0.367 0.143	0.971 **0.995**	0.055 0.003	0.963 **0.978**	0.033 0.022

FIB, tumor related fibrosis; AUG, augmentations; ACC, accuracy; SEN, sensitivity; SPE, specificity; F1, weighted F1-score; SD, standard deviation. Bold values indicate the highest value of the 2 metrics per model (with and without AUG).

**Table 6:** Mean and standard deviation of whole slide testing metrics for each sample, with and without augmentations.

WSI	AUG	ACC		SEN		SPE		F1	
		Mean	SD	Mean	SD	Mean	SD	Mean	SD
16	0 1	**0.999** 0.996	0.000 0.003	0.003 **0.167**	0.005 0.177	**0.999** 0.996	0.000 0.003	**0.999** 0.998	0.000 0.001
18	0 1	**0.996** 0.989	0.003 0.007	0.002 **0.094**	0.005 0.077	**0.997** 0.989	0.003 0.007	**0.997** 0.993	0.001 0.004
23	0 1	0.958 **0.961**	0.003 0.002	0.060 **0.127**	0.114 0.129	0.996 **0.997**	0.003 0.004	0.941 **0.947**	0.008 0.008
25	0 1	0.964 **0.966**	0.000 0.002	0.000 **0.117**	0.001 0.085	**0.999** 0.997	0.000 0.002	0.946 **0.954**	0.000 0.006
27	0 1	0.948 **0.988**	0.050 0.002	0.219 **0.249**	0.422 0.217	0.956 **0.995**	0.055 0.003	0.963 **0.986**	0.026 0.003

WSI, whole slide image; AUG, augmentations; ACC, accuracy; SEN, sensitivity; SPE, specificity; F1, weighted F1-score; SD, standard deviation. Bold values indicate the highest value of the 2 metrics per model (with and without AUG).

Upon analysis of this testing phase, models fine-tuned with augmentations consistently outperformed their non-augmented counterparts, as evidenced by the results in **Table 5**. Particularly, the ‘Interhospital’ model (Experiment 8) achieved the highest accuracy (0.982), sensitivity (0.256), and F1-score (0.978). Additionally, ‘Tumor Extended’ (Experiment 6) demonstrated the highest specificity (0.998). The Area Under the ROC Curve (AUC-ROC) further supported ‘Interhospital’ as the leading model (**Supplementary Figure 2**).

Examining patients individually, superior metrics were also evident in cases where augmentations were utilized (**Table 6**), aligning with the trends previously observed in the whole slide testing phase (**Table 5**). This observation emphasized the potential impact of augmentations on model performance, especially in scenarios involving domain changes and unseen images. Notably, patients 16 and 18 achieved the best accuracy and F1-score metrics, despite having low tumor patch content in their WSIs (0.03% and 0.35%, **Table 3**). This outcome indicated the models’ proficiency in identifying healthy tissue, offering potential benefits in the screening of patients with normal SLN biopsies.

Quantitative metrics analysis revealed that superior metrics were achieved without augmentations during model fine-tuning and patch-based testing (**Table 4**). This result was likely due to the low variability between the training and testing sets, sourced from the same WSIs. However, during whole slide testing, where domain changes occurred, models trained with augmentations demonstrated better performance (**Tables 5**, **6**). This underscored the importance of using models trained on diverse data when transitioning domains, enhancing their generalization capabilities.

**Figure 5:**
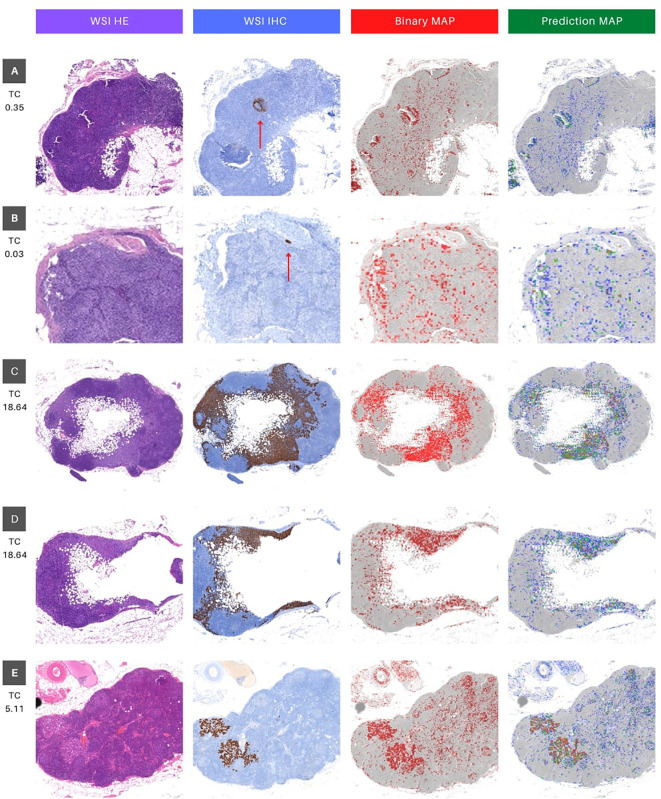
Visual interface for interpretation of classification results for WSI in whole slide testing. The figure consists of four columns, where the first column (WSI HE) presents the original whole slide image (WSI) in hematoxylin and eosin (HE), while the second column shows the ground truth marking with immunohistochemistry (IHC) for Cytokeratin (WSI IHC). The brown deposits inthe IHC marking represent cancer cells, and a red arrow is utilized as a visual aid to locate brown deposits in cases that may be too small to identify. The third column depicts a binary map (BIinary MAP), with red masking indicating patches with predictions above 0.5. Finally, the fourth column exhibits a heatmap that portrays the prediction probability (Prediction MAP), where red, green, and blue indicate high (above 0.9), medium (between 0.7 and 0.9), and low probability (between 0.5 and 0.7) predictions, respectively. The rows **(A–E)** represent different tissue sections of specific patients (16, 18, 25 and 27). Rows C and D represent different portions of WSI 25 (upper and lower, respectively). TC stands for tumor content and represents the percentage of tumor pixels in the WSI.

The qualitative metrics section served as a vital complement to the quantitative findings, offering valuable clinical insights. Cases with minimal tumor pixels, such as cases 16 and 18 (**Figure 5**), displayed distinct patterns from those with higher tumor counts. These cases exhibited tumor pixel rates of 0.03% and 0.35% across their WSIs, respectively (**Table 3**). The top-performing model, Interhospital, showed intersections with tumor regions in its predictions, but false positives were scattered throughout the images. Notably, false positives were also observed above areas of heterogeneous tissue with some degree of fibrosis in case 18 (**Figure 5**).

Furthermore, we evaluated the models’ performance in cases featuring a substantial proportion of tumor pixels per image. Specifically, we analyzed WSI case 25 (depicted in the fourth and fifth rows) and case 27 (shown in the final row of **Figure 5**).

These cases displayed tumor pixel ratios of 18.64% and 5.11%, respectively (refer to **Table 3**). A significant increase of at least 1600% in pixel percentage was observed when comparing cases with low and high tumor pixel percentages. This considerable rise in tumor pixels correlated with the tumor cell count, indicating an extended period of tumor growth from the initial metastatic cell invasion of the SLN to the biopsy. Consequently, this prolonged duration likely contributed to the distinct differences between tumor cells and normal cells visual phenotype, improving the model’s performance.

In the context of WSI 25, both tissue slices exhibited striking similarities between the ground truth area for IHC and the model’s predictions, as demonstrated in **Figure 5** cases 25.1 and 25.2. Conversely, in WSI 27, while the IHC and binary predictions showed a significant overlap (highlighted in red), the color-coded prediction map provided valuable insights (**Figure 5** case 27). Specifically, regions with the highest predicted probabilities (0.9 and above, indicated in red) almost entirely coincided with tumor areas. The remaining predictions, characterized by lower probabilities (below 0.9, depicted in green and yellow), played a vital role in distinguishing false positives from true positives (highlighted in red). This detailed analysis underscored the model’s accurate identification of tumor regions in cases with varying tumor pixel percentages, offering crucial information for clinical evaluation and diagnosis.

**Figure 6:**
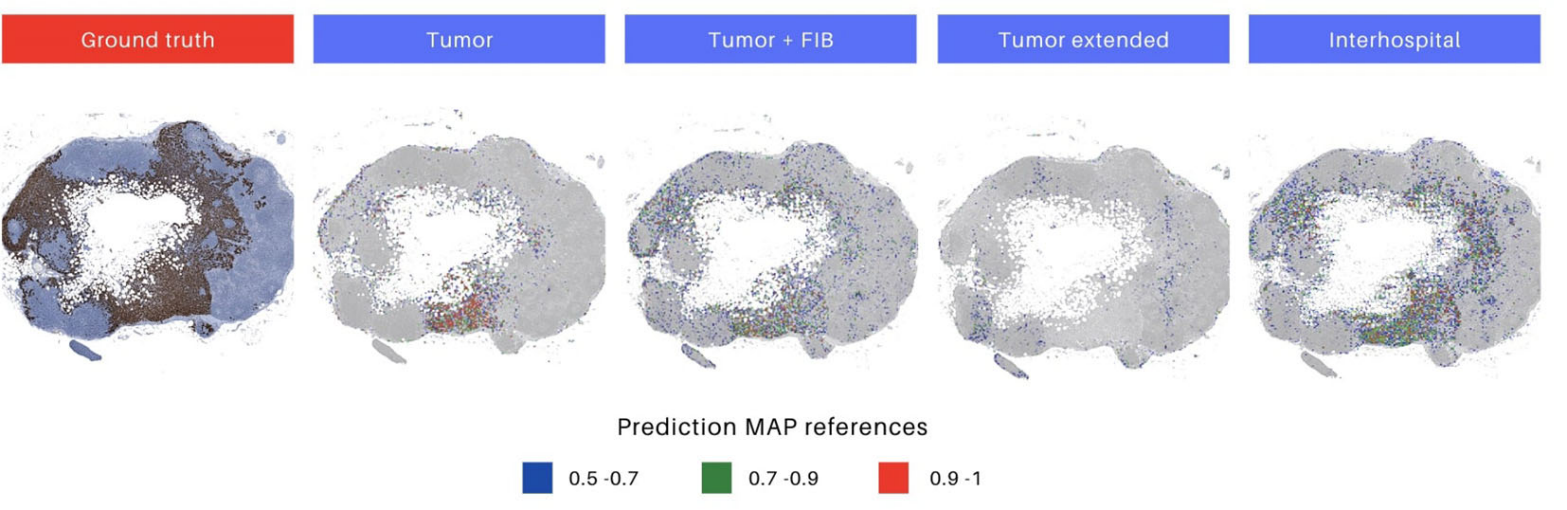
Differences in prediction map output for each model. The initial image represents the ground truth in immunohistochemistry (IHC), highlighting tumor areas. Images 2 to 5 depict prediction maps generated by individual models. The selected models for evaluation were those trained with augmentations, as they demonstrated superior performance. Heatmaps are constructed, utilizing red, green, and blue colors, to display the predicted probabilities for each patch. The color codes represent high (above 0.9), medium (0.7 to 0.9), and low probability (0.5 to 0.7) predictions, respectively.

Moreover, upon a meticulous inspection of the prediction maps generated by all models overlaid on the same image (**Figure 6**), the distinctive outcomes align with the quantitative metrics, as illustrated in the F1 score results in **Table 5** and the AUC ROC values presented in **Supplementary Figure 2**. Notably, the ‘Interhospital’ model consistently outperforms other models across all three scenarios, followed by ‘Tumor + FIB’. The preceding analysis provides two critical insights. First, it highlights the importance of the visual interface for comprehensively evaluating model performance. The interface enables direct visualization of model outputs in the context of the original histopathology images, conferring clinical meaning to quantitative metrics. Second, it underscores the significance of heterogeneity in the whole slide testing. As observed, the top performing models were fine-tuned on the most heterogeneous datasets. Specifically, the ‘Interhospital’ model was trained on samples from two distinct sources - locally generated data and the public Camelyon dataset. Meanwhile, the ‘Tumor + FIB’ model was trained on patches containing not only tumor cells, but also associated fibrous stromal tissue.

## 5 Discussion

This study demonstrates the effectiveness of transfer learning in fine-tuning ResNet50 models for accurately detecting metastatic regions in SLN biopsy images. By leveraging Cytokeratin markers in IHC images to generate masks, we created four distinct patch datasets for fine-tuning, incorporating both augmented and non-augmented samples. Patch-level testing revealed that the highest accuracy was achieved without augmentations, likely due to overlaps between test and training samples from the same WSIs. However, whole-slide testing showed improved performance with augmentations, underscoring the role of data variability in enhancing model generalization. Notably, incorporating additional variability—such as introducing samples from Camelyon into the Interhospital dataset—provided the optimal diversity level, leading to superior performance on unseen images. These findings suggest that supplementing data augmentation with diverse datasets that maintain domain specificity can further enhance model robustness.

Our results provide support for our three initial hypotheses. First, transfer learning with appropriate fine-tuning indeed achieved clinically relevant performance (accuracy up to 0.982) despite using only 25 WSIs for training, supporting that pretrained models can overcome data scarcity limitations. Second, the hybrid Interhospital dataset consistently outperformed models trained on single-institution data (accuracy improvements of 3-5%), demonstrating that combining institutional with public datasets enhances generalization. Third, our visual explainability tools were able to bridge the technical-clinical gap - pathologists could interpret the heatmaps and binary maps with minimal training, validating their utility in real diagnostic workflows. These findings collectively help demonstrate that our proposed approach addresses the key challenges of data efficiency, generalizability, and clinical adoption in computational pathology.

The observed variations in model training times reveal important insights about computational efficiency. Models trained on smaller datasets (‘Tumor’ and ‘Tumor + FIB’ with 23,900 samples) completed training significantly faster than those using larger collections. The ‘Interhospital’ dataset (104,107 samples) showed moderate training duration, while the extensive ‘Tumor Extended’ dataset (227,923 samples) required the longest processing time, confirming the expected scaling of computational most accurate model (patch-based testing: 0.881; whole-slide: 0.982 with augmentation), emphasizing that data diversity and quality outweigh sheer volume for metastatic detection tasks. This finding has important practical implications for clinical implementation, where both accuracy and computational efficiency are paramount.

We also noted a difference in performance between evaluating individual patches and entire slides. While the model consistently showed high specificity, meaning it was good at correctly identifying non-tumor samples, its sensitivity decreased when analyzing whole slides. This indicates a potential difficulty in detecting new tumor regions not seen during training. Data augmentation proved useful, as models trained with augmented data showed better tumor detection without sacrificing specificity. The best accuracy we achieved (0.98) was with an augmented model, which is comparable to results from prior studies classifying similar lymph node samples using GoogleNet (0.98), AlexNet (0.92), VGG16 (0.97), and FaceNet (0.96) (54–57). Other research on different types of oncology images, also using transfer learning and augmentations (including techniques like edge detection we didn’t explore), reported similar (VGG 19, ResNet 50, ResNet 152, MobileNet) and even higher accuracies (VGG 16, ResNet 101V2, DenseNet 169) (58).

Our evaluation metrics systematically assessed model performance across different testing conditions. When tested on previously unseen WSIs, models exhibited overall accuracy improvements, likely due to the higher proportion of non-tumor regions in WSIs, which enabled models to leverage their high specificity. However, the observed decrease in sensitivity highlights ongoing challenges in generalizing to new tumor data. The increase in weighted F1-score with additional training data suggests that models benefit from the larger proportion of healthy tissue in whole-slide images compared to tumor-enriched patch datasets. The ability to reliably identify non-tumor regions could facilitate computational pathology workflows by enabling automatic masking of healthy areas, allowing pathologists to focus on uncertain regions.

A key aspect of this work was creating a visual interface to improve clinical understanding. Overlaying identified metastatic areas onto the complete patient slides allowed pathologists to visually evaluate the model’s predictions within the original context. Our results demonstrated accurate tumor patch detection when tumor content was above 16%, consistent with our quantitative findings. Indeed, our patch-based heatmap overlay offered the ideal level of detail for pathologists to differentiate zones of tumor development in the predictions. As noted, larger metastatic deposits tend to exhibit greater cellular diversity due to longer growth periods and potential variations in oxygen and nutrient access, leading to distinct morphologies within the same cluster (59).

Additionally, our approach offers a time-efficient alternative to traditional IHC procedures. Generating IHC-stained slides from biopsy tissue can take up to 16 hours (60, 61), whereas our models process an HE-stained WSI in approximately 18 minutes—a 98% reduction in processing time. By enabling rapid, automated identification of tumor and healthy regions, this approach has the potential to streamline computational pathology workflows and reduce clinician workload. However, challenges persist in cases with low tumor cell content or poorly differentiated morphology—such as Cases 16 and 18—where tumor cells closely resemble normal tissue. In addition to the lack of clear differentiation, these tumor regions covered a relatively small surface area, resulting in fewer representative patches with this morphology in the training data. This raises an important question: is the issue primarily due to poor differentiation, or is it the limited representation of such cases in the training dataset? To explore this, future studies should focus on datasets containing a sufficient number of poorly differentiated tumor samples alongside normal tissue. Despite these limitations, the model’s ability to accurately exclude healthy tissue and flag uncertain regions for expert review highlights its time-saving potential compared to manual IHC slide analysis.

The clinical implications of this work extend beyond technical performance metrics. Our framework demonstrates how computational pathology can be strategically adapted to institutional settings through three key principles: leveraging transfer learning to overcome data limitations, curating hybrid datasets that balance domain specificity with diversity, and developing interpretation tools that align with pathologists’ diagnostic workflows. This approach not only achieves diagnostic accuracy comparable to manual assessment but does so while respecting the practical constraints of clinical environments, where computational resources and annotation bandwidth are often limited.

Looking forward, two parallel pathways emerge for advancing this research. First, technical refinements should focus on improving detection of diagnostically challenging cases, particularly micrometastases and tumors with ambiguous morphology. Second, clinical validation studies must establish how best to integrate such systems into real-world workflows, including optimal division of labor between AI and pathologists. The interpretability tools developed here provide a foundation for this transition, enabling collaborative decision-making where computational efficiency complements human expertise. Together, these directions promise to accelerate the translation of AI-assisted pathology from research laboratories to routine clinical practice.

## 6 Conclusions

This study validates three fundamental advancements in computational pathology for metastatic detection. First, we demonstrate that transfer learning with targeted fine-tuning can achieve clinical-grade accuracy using limited training data, overcoming establish that intuitive visualization tools effectively bridge the gap between computational outputs and clinical interpretation, facilitating pathologist adoption.

The success of our framework stems from its synergistic approach: (1) optimizing data efficiency through strategic transfer learning, (2) improving robustness via multi-source dataset integration, and (3) ensuring clinical relevance through interpretable visual analytics. These innovations collectively address the tripartite challenge of data scarcity, domain generalization, and workflow integration that has hindered widespread adoption of AI in pathology.

While demonstrating significant improvements over conventional methods, two key opportunities emerge for future research: enhancing detection of minimal residual disease and improving performance on morphologically ambiguous cases. Clinical translation of this validated pipeline could transform metastatic screening protocols by combining the efficiency of AI with pathological expertise, potentially establishing new standards for rapid, accurate lymph node assessment. The principles developed here may extend to other histopathological applications where data limitations and clinical interpretability remain persistent challenges.

## Data Availability

The WSI HE images and ground truth IHC from the Pathology Laboratory of Hospital Clinic Barcelona are inaccessible to the public to uphold individual privacy in compliance with the European General Data Protection Regulation. However, WSI HE images and ground truth masks from the Camelyon 16 dataset are accessible to the public via this site: https://camelyon16.grand-challenge.org/Data/. Requests to access these datasets should be directed to Geert Litjens, Geert.Litjens@radboudumc.nl.

## Footnotes

1
https://www.clinicbarcelona.org/.

2 See Section 6.1: Ethics Approval and Consent to Participate.

3
https://pypi.org/project/labelme/.

4 Using Fastai (1.0.61), Pytorch (1.0.0), and Openslide (1.2.0) packages.

5 IMDRF/SaMD WG/N41; Software as a Medical Device (SaMD): Clinical Evaluation.
