# Supplementary material for: AI-augmented pathology: the experience of transfer learning and intra-domain data diversity in breast cancer metastasis detection
Source: Front Oncol. 2025 Jun 11;15:1598289. doi: 10.3389/fonc.2025.1598289 (PMC12187835; doi:10.3389/fonc.2025.1598289)
Supplement: Supplementary file 1 [file DataSheet1.pdf]

Appendix

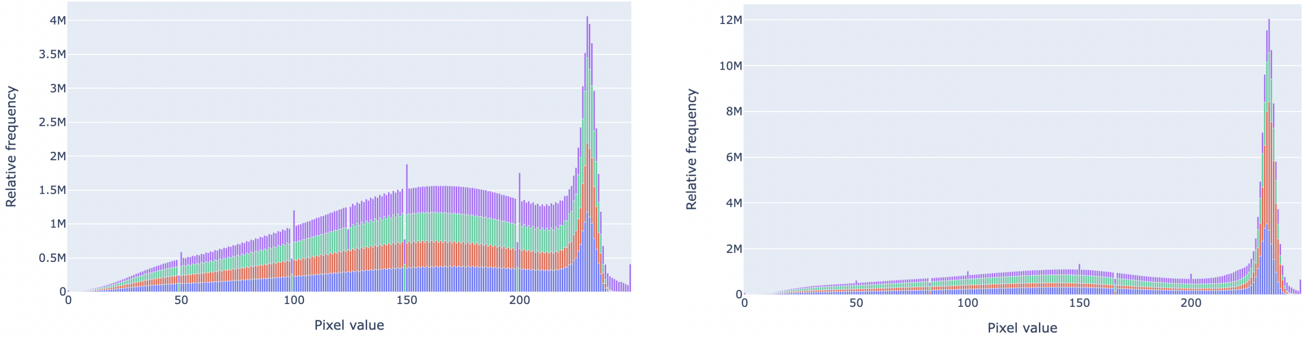

Supplementary Figure 1: **Histograms of Pixel Value Distributions Across Datasets Highlighting Inter-Dataset Variability.** This figure presents histograms illustrating the pixel value distributions within the patch datasets used for training the metastasis detection models. The histograms are generated using 250 bins to provide a detailed view of the frequency of pixel intensities. The left panel displays the distributions for positive (tumor-containing) patches, and the right panel shows the distributions for negative (non-tumor) patches. Each colored line represents a different dataset: blue for the 'Tumor' dataset, which contains only tumor regions from the Hospital Clinic Barcelona (HCB) samples; red for the 'Tumor + FIB' dataset, which includes tumor and tumor-associated fibrosis regions from HCB samples; green for the 'Tumor extended' dataset, an expanded set of tumor regions also from HCB samples; and purple for the 'Interhospital' dataset, a mix of 50% HCB 'Tumor' patches and 50% patches from the Camelyon16 dataset. These histograms highlight the inherent variability in pixel intensities across datasets. The x-axis represents the pixel value, and the y-axis represents the relative frequency of pixels within each bin.

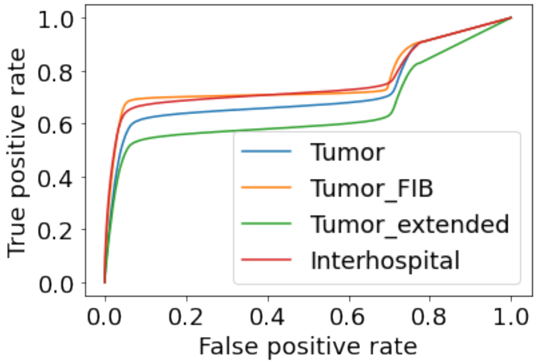

Supplementary Figure 2: **ROC Curves Generated for Each Trained Model that Incorporates Augmentations**. This figure displays Receiver Operating Characteristic (ROC) curves for the fine-tuned ResNet50 models. The ROC curves plot the true positive rate (sensitivity) against the false positive rate (1 - specificity) across various classification thresholds. Each curve corresponds to a model trained on a specific dataset with data augmentation: blue represents the model trained on the 'Tumor' dataset, orange on 'Tumor + FIB', green on 'Tumor extended', and red on the 'Interhospital' dataset. These datasets were constructed from HE stained WSIs, with the 'Interhospital' dataset including external data from the Camelyon16 challenge. The models were fine-tuned using a transfer learning approach.
